# Global peak left atrial longitudinal strain assessed by transthoracic echocardiography is a good predictor of left atrial appendage thrombus in patients in sinus rhythm with heart failure and very low ejection fraction – an observational study

**DOI:** 10.1186/s12947-020-00188-0

**Published:** 2020-02-15

**Authors:** Jacek Kurzawski, Agnieszka Janion-Sadowska, Lukasz Zandecki, Lukasz Piatek, Dorota Koziel, Marcin Sadowski

**Affiliations:** 1 Swietokrzyskie Cardiology Centre, Kielce, Poland; 2 The Jan Kochanowski University, ul. Stefana Zeromskiego 5, 25-001 Kielce, Poland

**Keywords:** Peak left atrial longitudinal strain, Left atrial appendage thrombus, Heart failure with reduced ejection fraction, Sinus rhythm

## Abstract

**Background:**

Peak left atrial longitudinal strain (PALS) can help identify left atrial appendage thrombus (LAAT) in patients with atrial fibrillation. Nevertheless, few studies have been performed in patients in sinus rhythm without established indications for anticoagulation but with increased risk of LAAT, such as heart failure (HF) with severe left ventricular systolic dysfunction patients. The primary aim of this study was to identify clinical and transthoracic echocardiography predictors of LAAT in HF patients with very low left ventricular ejection fraction and sinus rhythm. The secondary objective was to analyze frequencies and predictors of a composite clinical endpoint of death or hospitalization for ischemic stroke.

**Methods:**

We included 63 patients with HF, left ventricular ejection fraction < 25%, sinus rhythm at presentation, no history of atrial fibrillation, and without any established indications for anticoagulation. We determined whether clinical and transthoracic echocardiography parameters, including left atrial strain analysis, predicted LAAT. Transesophageal echocardiography was performed in all patients. When LAAT was detected, anticoagulation was recommended. The participants were followed for a median of 28.6 months (range 4–40) to determine the composite endpoint.

**Results:**

LAAT was found in 20 (31.7%) patients. Global PALS was the best independent predictor of LAAT in univariate and multivariate logistic regression analyses (Gini coefficient 0.65, area under the receiver-operating characteristic curve 0.83). A global PALS value below 8% was a good discriminator of LAAT presence (odds ratio 30.4, 95% CI 7.2–128, *p* <  0.001). During follow-up, 18 subjects (28.6%) reached the composite clinical endpoint. CHA_2_DS_2_-VASc score, use of angiotensin-converting-enzyme inhibitors or angiotensin receptor blockers, and body surface area were significant predictors for the composite endpoint of death or hospitalization for ischemic stroke in the multivariate regression model.

**Conclusions:**

LAAT was relatively common in our group of HF patients and PALS has shown prognostic potential in LAAT identification. Further research is needed to determine whether initiation of anticoagulation or additional screening supported by PALS measurements will improve clinical outcomes in these patients.

## Background

Heart failure (HF) with reduced left ventricular (LV) ejection fraction (LVEF) is associated with an increased risk of ischemic stroke and mortality, in both sinus rhythm (SR) and in atrial fibrillation (AF). The risk of stroke in HF patients appears to be directly related to the degree of LV systolic dysfunction and increases when the LVEF < 30% [1]. The risk may be even higher in subjects with extremely low LVEF values (< 15%) [2]. However, the overall stroke rate that may be associated with HF is too low to justify anticoagulation in all patients, even in those with reduced LVEF [3]. Despite limited evidence, the current opinion holds that anticoagulation should be considered in HF patients with reduced LVEF and previous thromboembolism or with newly diagnosed intracardiac thrombus [4].

Many imaging modalities are employed to detect intracardiac thrombi, each of which has limitations. Transesophageal echocardiography (TEE) is a semi-invasive procedure. Computed tomography entails substantial radiation exposure and potential nephrotoxicity due to intravenous contrast agents. Magnetic resonance is costlier and is not always readily available. By contrast, peak atrial longitudinal strain (PALS) alone is reported to be useful for functional analysis of the left atrium (LA) [5, 6] and in prediction of left atrial appendage thrombus (LAAT) or reduced LA appendage velocity in patients with AF [5, 7, 8]. There is insufficient data on PALS performance in patients with SR with an increased risk of LAAT, such as those with HF with severe LV systolic dysfunction. Two transthoracic echocardiography (TTE) techniques are currently used for functional analysis of LA: speckle tracking echocardiography and tissue doppler imaging (TDI). Both use standard two-dimensional images for the analysis of myocardial deformation and have some well-acknowledged advantages and disadvantages [9]. LA strain assessment techniques are fairly simple with high feasibility and reproducibility [8, 10]. In patients with SR, both PALS in the conduit phase and peak atrial contraction strain (PACS) analyses are feasible [11, 12]. Nevertheless, it is not known if PALS predicts LAAT in patients in SR.

The main objective of this study was to identify predictors of LAAT presence in patients with HF, very low EF and in SR. The additional objective was to analyze the clinical outcomes in these patients.

## Methods

### Study design

The study was conducted at Swietokrzyskie Cardiology Centre in a large tertiary hospital in Poland.

The primary aim of this study was to determine whether any clinical or TTE data, including PALS, are independent predictors of LAAT in patients with very low EF, with SR at presentation, without history of AF, and not receiving anticoagulation for other indication. The secondary objective was to analyze the frequencies and predictors of a composite clinical endpoint of death or hospitalization for ischemic stroke.

All adult patients were eligible for study enrolment if they had HF diagnosis documented in their medical history, SR on electrocardiography (ECG), and reduced LVEF < 25% calculated with the biplane Simpson’s method using the apical four chamber (A4C) and apical two chamber (A2C) views during TTE examination. Exclusion criteria were as follows:

- Documented history of AF, atrial high-rate episodes (AHRE) recorded by cardiac implantable electronic devices (CIED), or any other indication for anticoagulation or receiving anticoagulation within 30 days prior to the enrolment.
- Potentially confounding valvular heart disease (any mitral stenosis, moderate or severe aortic stenosis or regurgitation, severe mitral or tricuspid regurgitation).
- Active cancer.
- Inability to provide informed consent.

In patients with acute HF, clinical stabilization was necessary before study enrolment. Patients with systolic blood pressure (BP) < 90 mmHg or > 140 mmHg, diastolic BP > 100 mmHg, or heart rate (HR) < 50 or > 100 beats per minute were not enrolled until BP and HR were normalized.

### Data collection

Collected clinical data included age, gender, HF etiology, hypertension, diabetes, chronic kidney disease, history of previous ischemic stroke or transient ischemic attack (TIA), and active cancer. The CHA_2_DS_2_-VASc score was calculated for each patient. Height and weight were measured by a trained observer with the patient wearing in light clothing. Body mass index (BMI) and body surface area (BSA) were calculated. In every participant, TTE followed by TEE were performed on the same day. The participants were followed in prospective cohorts for death and hospitalizations due to ischemic strokes to determine the secondary objective of the study. Data on death and hospitalizations for ischemic strokes were obtained from the official records of the National Health Fund. Written informed consent was obtained from all participants. The study protocol conformed to the ethical guidelines of the 1975 Declaration of Helsinki. The study was approved by the appropriate bioethics committee.

### Transthoracic echocardiography

TTE was performed using the General Electric Vivid E9 device and a sector array M5S (2.5–3.5 MHz) transducer. HR was measured at the time of TTE. Images were acquired to measure LA diameter, LA area, LA volume index (LAVI), and LVEF. LA diameter was measured at the end of the left ventricle systole in the parasternal long axis view. LA area and LAVI were measured in the A4C and A2C views at end systole. Mitral flow velocities with E/A ratios (the ratio of early to late transmitral diastolic velocities) were measured using pulse-wave Doppler, TDI was used to measure e` values (pulsed-wave TDI-derived mitral annular early diastolic velocity), and E/e` values were then calculated. All TEE and TTE measurements were performed by a single echocardiography expert.

### Transesophageal echocardiography

The General Electric Vivid E9 device and a GVT-D (3–8 MHz) transducer were used to perform TEE to allow visualization of left atrial appendage and detection of LAAT. LAAT was diagnosed when a fixed or mobile echogenic mass could be clearly differentiated from the wall of the LA or LA appendage. Patients in whom LAAT was identified had anticoagulation treatment initiated and prescribed at discharge unless contraindicated – the choice among antithrombotic regimens was made at the discretion of the treating physician.

### Left atrial strain analysis

**Fig. 1:**
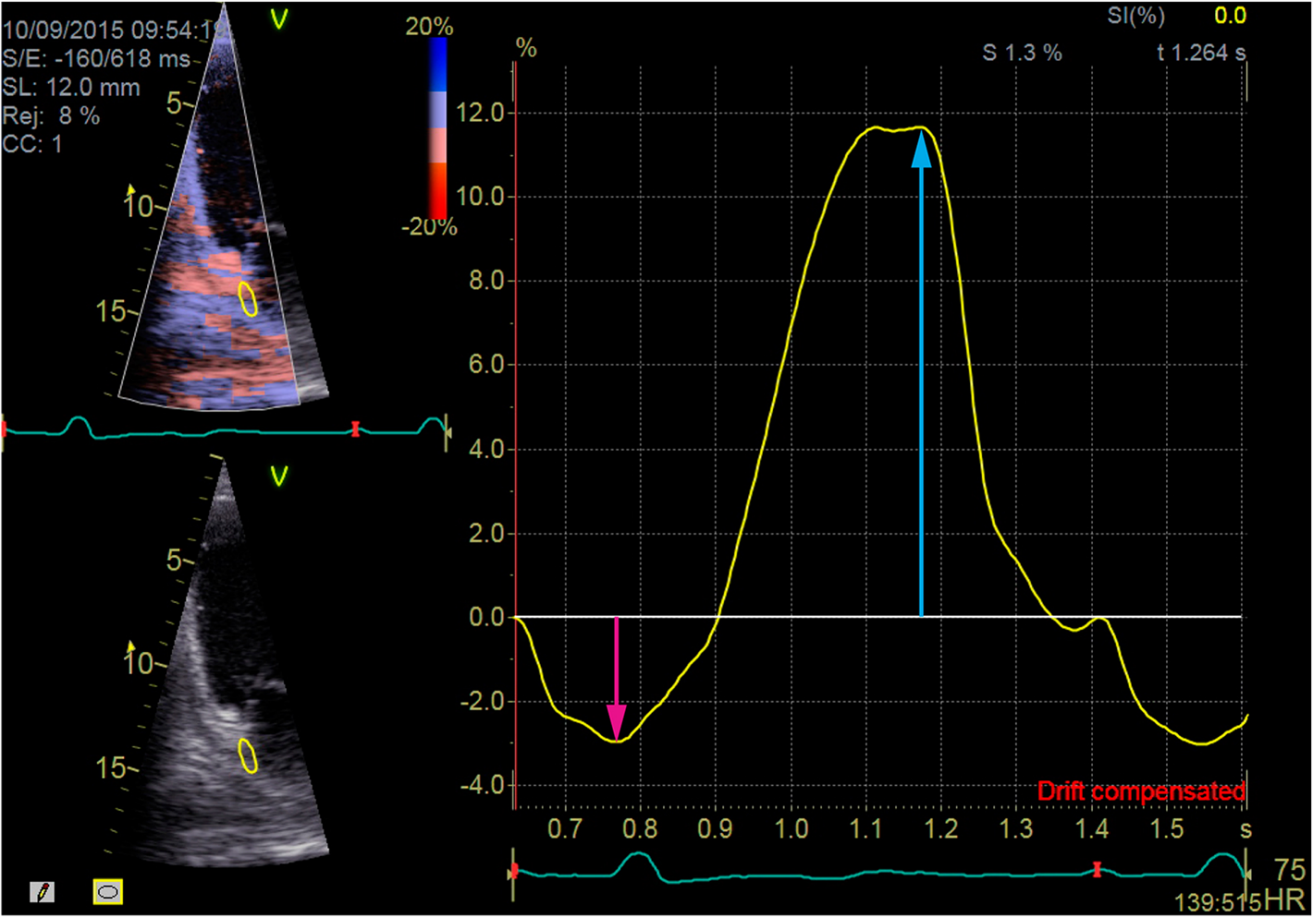
A sample of strain curves Q-analysis in the region of interest. Pink arrow indicates contractile strain (PACS) and blue arrow indicates reservoir strain (PALS) measurements

TDI LA images were obtained with stable ECG tracing in the A4C view (with an interatrial septum and LA lateral wall visible) and the A2C view (attempting to visualize LA inferior and anterior walls). The view angle was obtained by positioning the analysis segment of the LA wall as much as possible along the direction of the ultrasound wave propagation. TDI was performed at a frame rate of over 100 frames per second. TDI echocardiograms including potential sites for strain analysis within the region of interest during at least three cardiac cycles were recorded. TDI recordings were necessary for off-line Q-Analysis as required by the software (General Electric EchoPac workstation, version 112; upgrade BT12). PALS and PACS in six selected regions of interest were analyzed: 1 - within intra-atrial septum at its base and close to the fossa ovalis, 2 - at its superior portion over the fossa ovalis, 3 - within the LA basal lateral wall, 4 - medial lateral wall, 5 - basal inferior wall, and 6 - medial inferior wall. The LA anterior wall was excluded from analysis because of technical difficulties in acquiring accurate images. The P wave was used as the reference point. The negative peak reflecting the extent of myocardial fiber shortening during atrial contraction was evaluated as the lowest value below the baseline and was described as LA contractile strain – PACS. The positive peak strain - PALS, reflecting LA stretching during the LA reservoir phase was recorded as the most positive elevation of the strain curve after the R wave of the QRS complex (Fig. 1). Three measurements from consecutive heart cycles were averaged. Global PALS and global PACS were calculated by averaging respective values observed in all six analyzed LA segments. Recordings for offline strain analyses were made by a single echocardiography expert in all patients at the time of initial TTE. PALS and PACS measurements were performed at the end of the study enrolment by one echocardiography expert who was blinded to patient data.

### Statistical analysis

Continuous variables are expressed as means ± standard deviation or medians and interquartile ranges, depending on their distribution. Categorical variables are expressed as counts and percentages. The significance of the differences between the study groups was determined using the Student t-test or Mann-Whitney U test for continuous variables and χ^2^ tests for categorical variables. A two-sided *p*-value ≤0.05 was considered significant. Unconditional univariate logistic regressions were performed to investigate the relationships between each clinical or echocardiographic variable and the risk of LAAT. Next, a multivariate logistic regression model was built using a stepwise forward selection approach. Another logistic regression model was built to identify predictors of a composite clinical endpoint of death or hospitalization for ischemic stroke. Wald chi-square statistic and likelihood ratio tests were used to calculate *p*-values. The predictive values of selected variables were assessed using receiver-operating characteristic (ROC) analyses. The Youden index was used to determine optimal cut-off values from the ROC curves.

To assess reliability and reproducibility of PALS measurements, 10 sets of TDI TTE recordings of 10 patients from the current study were randomly chosen. PALS analyses were repeated by the echocardiography expert who performed the initial measurements and who was experienced in TDI PALS analysis (over 500 patients) and independently by a second echocardiography expert who was only moderately experienced in the PALS analysis using TDI (around 50 patients). The observers were blinded to the patient data. The analysis involved off-line measurements of already acquired data. The observers were allowed to freely choose from several cardiac recorded cycles for each patient. The Bland-Altman method was used to estimate mean differences of intra- and inter-observer variability and the limits of agreement. Intra- and inter-observer reproducibility of PALS measurements was quantified using the intra-class correlation coefficients (ICC), which were generated using one-way ANOVA models. Standard errors of measurement (SEM) were calculated as a root mean square error [13].

Calculations and statistical analyses were performed using Dell Statistica (data analysis software system), version 13. Dell Inc. (2016).

## Results

### Study population

From June 2015 to April 2018, a total of 258 patients with SR, HF and LVEF < 25% were screened for eligibility to enter the study. Sixty-three patients who met the selection criteria were included. Of these, 40 (63.5%) had a CIED and 24 (38.1%) underwent 24- or 48-h Holter ECG monitoring during index hospitalization.

Clinical and echocardiographic data are presented in Table 1. LAAT was detected using TEE in 20 (31.7%) patients. There were no significant differences with regard to the baseline clinical data between patients with and without LAAT. However, the groups differed with regard to some baseline echocardiographic data including LAVI, which was higher in the group with LAAT (68.6 ml/m^2^ (± 9.9) vs. 59.1 ml/m^2^ (± 13.7), *p* = 0.01), and global PALS and global PACS, which were lower in the group with LAAT (7.2% (± 2.1) vs. 9.7% (± 1.3), *p* <  0.001 and − 3.6% (± 1.1) vs. -4.2% (± 1.2), *p* = 0.04, respectively). In terms of the follow-up data there were no significant differences. There were more hospitalizations for ischemic strokes in the patients with LAAT but the incidence was low and the difference was not statistically significant (2 patients (10%) in the group with LAAT and 1 patient (2.3%) in the group without LAAT, *p* = 0.49). A comparison of the baseline characteristics of patients with and without LAAT is displayed in Table 1 and follow-up data is presented in Table 2.

**Table 1:** Baseline characteristics of patients with and without LAAT confirmed on TEE

	All patients (*n* = 63)		With LAAT (*n* = 20, 31.7%)		Without LAAT (*n* = 43, 68.3%)		*p*-value
Age (years)	61.9	(±10.9)	62.8	(±9.9)	61.5	(±11.4)	0.67
Male sex	60	(95.2%)	20	(100%)	40	(93%)	0.57
Heart rate (bpm)	77.3	(±10.9)	76.0	(±10.8)	78.0	(±11.1)	0.51
BMI (kg/m^2^)	26.2	(±4.5)	26.1	(±4.7)	26.3	(±4.5)	0.83
BSA (m^2^)	1.9	(±0.2)	1.9	(±0.2)	1.9	(±0.2)	0.4
Ischaemic HF aetiology	33	(52.4%)	13	(65%)	20	(46.5%)	0.17
Hypertension	34	(54%)	11	(55%)	23	(53.5%)	0.91
Diabetes	21	(33.3%)	5	(25%)	16	(37.2%)	0,34
Chronic kidney disease	28	(44.4%)	9	(45%)	19	(44.2%)	0.95
History of stroke or TIA	7	(11.1%)	3	(15%)	4	(9.3%)	0.81
CHA_2_DS_2_-VASc score	3	(2–4)	3	(2–5)	3	(2–4)	0.65
NYHA class							0.59
I	0	(0%)	0	(0%)	0	(0%)	
II	7	(11.1%)	3	(15%)	4	(9.3%)	
III	34	(54%)	9	(45%)	25	(58.1%)	
IV	22	(34.9%)	8	(40%)	14	(32.6%)	
Aspirin	52	(82.5%)	17	(85%)	35	(81.4%)	0.99
Other antiplatelet agent	11	(17.5%)	6	(30%)	5	(11.6%)	0.15
Statin	39	(61.9%)	12	(60%)	27	(62.8%)	0.83
Diuretic	62	(98.4%)	19	(95%)	43	(100%)	0.69
Beta-blocker	58	(92.1%)	18	(90%)	40	(93%)	0.93
ACE-I or ARB	43	(68.3%)	16	(80%)	27	(62.8%)	0.17
MRA	53	(84.1%)	16	(80%)	37	(86.1%)	0.81
Calcium channel blocker	2	(3.2%)	0	(0%)	2	(4.7%)	0.84
Digoxin	2	(3.2%)	0	(0%)	2	(4,7%)	0.84
LA diameter (cm)	5.0	(±0.4)	5.0	(±0.3)	5.1	(±0.5)	0.6
LAA (cm^2^)	31.3	(±7.4)	31.1	(±5.5)	31.4	(±8.1)	1
LAVI (ml/m^2^)	62.1	(±13.3)	68.6	(±9.9)	59.1	(±13.7)	0.01
LVEF (%)	19.2	(±4.1)	18.9	(±5)	19.4	(±3.7)	0.99
E/A	2.4	(±1.1)	2.7	(±1.3)	2.3	(±1)	0.28
E/e`	24.2	(±8.4)	23.6	(±8.3)	24.5	(±8.5)	0.66
Global PALS (%)	8.9	(±2)	7.2	(±2.1)	9.7	(±1.3)	< 0.001
Global PACS (%)	−4.0	(±1.2)	−3.6	(±1.1)	−4.2	(±1.2)	0.04

Values are means (± standard deviations), median (interquartile range) or counts (%)

*LAAT* Left atrial appendage thrombus, *TEE* Transesophageal echocardiography, *bpm* Beats per minute, *BMI* Body mass index, *BSA* Body surface area, *HF* Heart failure, *TIA* Transient ischemic attack, *NYHA* New York Heart Association, *ACE-I* Angiotensin-converting-enzyme inhibitors, *ARB* Angiotensin receptor blockers, *MRA* Mineralocorticoid receptor antagonist, *LA* Left atrium, *LAA* LA area, *LAVI* LA volume index, *LVEF* Left ventricular ejection fraction, E/A The ratio of early to late transmitral diastolic velocities, e’ - pulsed-wave TDI-derived mitral annular early diastolic velocity, *PALS* Peak atrial longitudinal strain, *PACS* Peak atrial contraction strain

**Table 2:** Follow-up data of patients with and without LAAT confirmed on TEE

	All patients (*n* = 63)		With LAAT (*n* = 20, 31.7%)		Without LAAT (*n* = 43, 68.3%)		*p*-value
Death	16	(25.4%)	5	(25%)	11	(25.6%)	0.96
Hospitalization for ischemic stroke	3	(4.8%)	2	(10%)	1	(2.3%)	0.49
Composite endpoint ^a^	18	(28.6%)	7	(35%)	11	(25,6%)	0.44

Values are counts (%). Abbreviations as in Table 1. ^a^Composite clinical endpoint of death or hospitalization for ischemic stroke

### Primary objective - LAAT predictors

In univariate logistic regression analyses, global PALS was shown to have the highest value in predicting LAAT (OR 0.43 (95% CI – 0.29 - 0.65), *p* <  0.001, Gini coefficient 0.65). Among all other available clinical and echocardiographic variables, PALS had the lowest odds ratio (inverse relationship) and the highest Gini coefficient (Table 3).

**Table 3:** Ranking of potential predictors for LAAT incidence in the study population based on univariate regression analyses. Sorted by the highest predictive value with Gini coefficients

	OR	−95% CI OR	95% CI OR	*p*-value	Gini coefficient
Global PALS (%)	0.43	0.29	0.65	< 0.001	0.65
LAVI (ml/m^2^)	1.06	1.01	1.11	0.01	0.44
Global PACS (%)	1.67	1.02	2.75	0.04	0.36
Other antiplatelet agent	3.27	0.86	12.39	0.08	0.18
Ischaemic HF etiology	2.14	0.71	6.4	0.18	0.18
ACE-I or ARB	2.37	0.67	8.34	0.18	0.17
Diabetes	0.56	0.17	1.84	0.34	0.12
BSA (m^2^)	0.26	0.01	5.73	0.4	0.11
E/A	1.31	0.80	2.16	0.28	0.1
LA diameter (cm)	0.71	0.2	2.51	0.59	0.1
E/e`	0.99	0.92	1.05	0.66	0.1
CHA_2_DS_2_-VASc score	1.02	0.75	1.39	0.9	0.08
BMI (kg/m2)	0.99	0.88	1.11	0.82	0.07
Heart rate (bpm)	0.98	0.94	1.03	0.5	0.06
History of stroke or TIA	1.72	0.35	8.54	0.51	0.06
MRA	0.65	0.16	2.62	0.54	0.06
Aspirin	1.3	0.30	5.51	0.73	0.04
NYHA class					0.03
II	1.4	0.48	4.08	0.54	
III	0.67	0.31	1.45	0.31	
IV	1.07	0.48	2.38	0.88	
Beta-blocker	0.68	0.10	4.4	0.68	0.03
Statin	0.89	0.30	2.64	0.83	0.03
Age (years)	1.01	0.96	1.06	0.67	0.02
Hypertension	1.06	0.37	3.08	0.91	0.02
LVEF (%)	0.97	0.85	1.11	0.66	0.01
Chronic kidney disease	1.03	0.36	3.00	0.95	0.01
LAA (cm^2^)	0.99	0.92	1.07	0.85	0

*OR* Odds ratio, *CI* Confidence interval. Other abbreviations as in Table 1

The first step of the multivariate logistic regression model building was the inclusion of global PALS. Following the first step, none of other available variables significantly improved the model. Global PALS remained an independent predictor for LAAT after adjustment for other applicable variables.

**Fig. 2:**
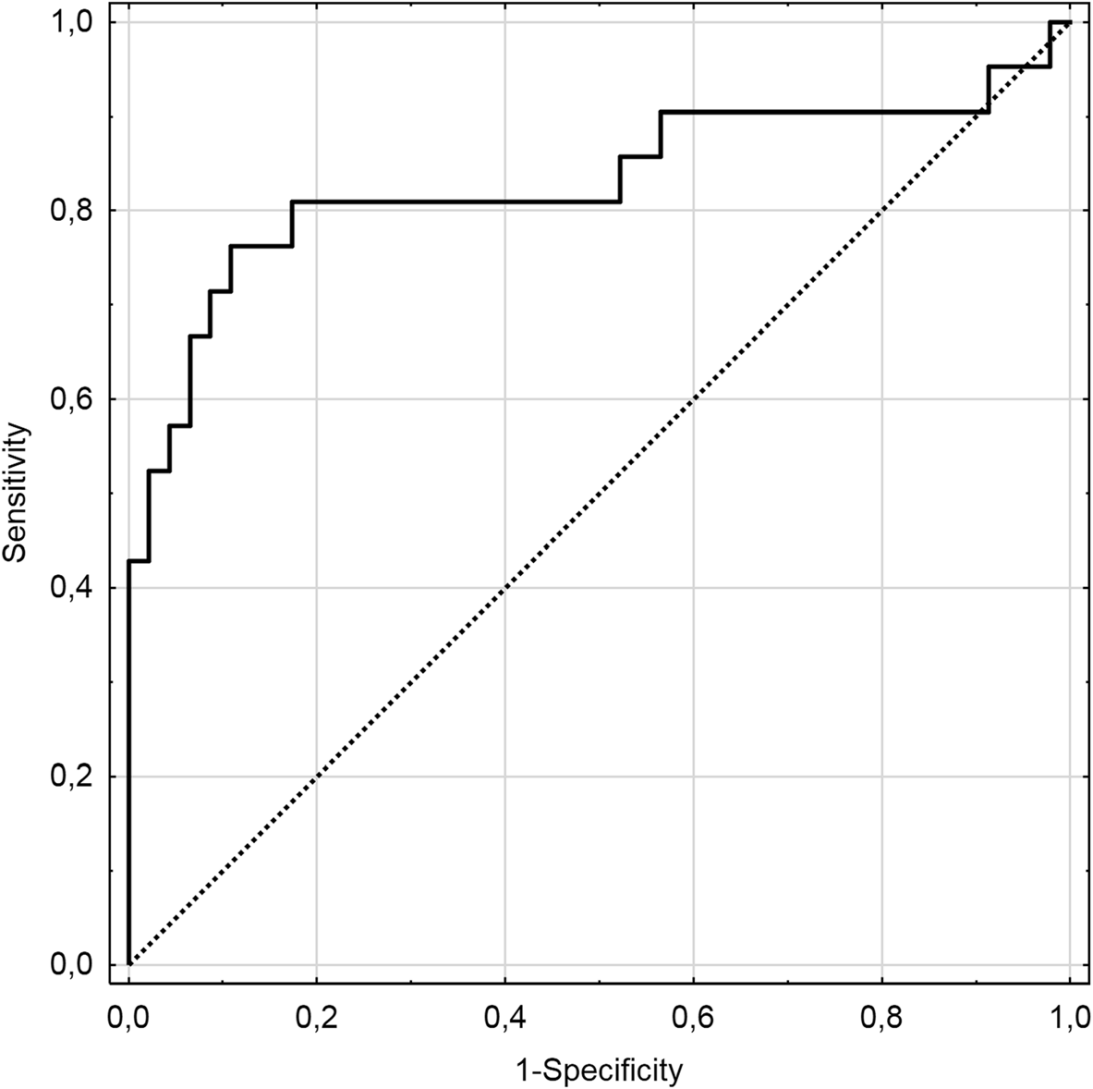
Receiver operating characteristic curve for predicting left atrial appendage thrombus from global PALS (solid line). Area under the receiver-operating characteristic curve (AUC) for global PALS equalled 0.83

The area under the ROC curve (AUC) for global PALS was 0.83 (Fig. 2). The optimal cut-off point from the ROC of global PALS was 7.7%. Given the next unique value of 8.43%, the best cut-off point for this subset could be anywhere between 7.7 and 8.43%; this suggests that the global PALS value of around 8% serves as a good discriminator of LAAT presence in this patient group. Dichotomized global PALS gave a calculated odds ratio of 30.4 (95% CI – 7.2 - 128) for LAAT presence if global PALS was < 8% (*p* <  0.001).

### Reliability and reproducibility of PALS measurements

The mean difference of intra-observer variability for PALS measurements was − 0.1 and the limits of agreement were − 0.8 and 0.6. The mean difference of inter-observer variability was − 0.6 and the limits of agreement were − 3.0 and 1.9. ICC for intra- and inter-observer concordance for PALS measurements were 0.98 (95% CI 0.92–0.99) and 0.77 (95% CI 0.31–0.94), respectively. Intra- and inter-observer SEM were 0.26 (95% CI 0.15–0.37) and 0.92 (95% CI 0.52–1.32), respectively.

### Secondary objective - composite endpoint of death or hospitalization for ischemic stroke

Patients with a confirmed diagnosis of LAAT were discharged and prescribed the following anticoagulants: 2 patients on acenocoumarol, 4 on warfarin, 8 on low molecular weight heparin, 3 on rivaroxaban, 2 on dabigatran, and 1 on apixaban. At a median follow-up of 28.6 months (range 4–40), 18 (28.6%) subjects met the composite clinical endpoint of death or hospitalization due to ischemic stroke (Table 2). The results of univariate regression models for prediction of secondary composite endpoint are presented in Table 4. Neither presence of LAAT nor PALS values were significant predictors of the composite endpoint of death or hospitalization for ischemic stroke. CHA_2_DS_2_-VASc score, use of ACE-I (angiotensin-converting-enzyme inhibitors) or ARB (angiotensin receptor blockers), and BSA were significant predictors for the composite endpoint of death or hospitalization for ischemic stroke in the multivariate regression model (Table 5).

**Table 4:** Results of the univariate regression models for prediction of secondary composite endpoint of death or hospitalization for ischemic stroke. Sorted by Gini coefficients

	OR	−95% CI OR	95% CI OR	*p*-value	Gini coefficient
CHA_2_DS_2_-VASc score	1.51	1.07	2.12	0.02	0.38
Age (years)	1.07	1.00	1.12	0.06	0.37
BSA (m^2^)	0.03	0.001	1.01	0.05	0.37
Chronic kidney disease	3.63	1.14	11.5	0.03	0.31
NYHA class					0.31
II	0.50	0.12	2.18	0.36	
III	0.79	0.31	2.01	0.61	
IV	2.52	0.98	6.47	0.05	
Heart rate (bpm)	0.95	0.91	1.01	0.08	0.28
ACE-I or ARB	0.32	0.1	1.02	0.05	0.26
Diabetes	2.75	0.88	8.56	0.08	0.23
Ischaemic HF etiology	2.29	0.73	7.16	0.16	0.2
History of stroke or TIA	4.00	0.80	20.1	0.09	0.16
LA diameter (cm)	1.16	0.32	4.15	0.82	0.12
LAAT	1.57	0.50	4.93	0.44	0.1
Aspirin	2.00	0.39	10.3	0.41	0.09
LAVI (ml/ m^2^)	1.01	0.97	1.05	0.74	0.09
LVEF (%)	0.96	0.84	1.09	0.50	0.09
MRA	0.54	0.13	2.19	0.39	0.09
BMI (kg/m2)	1.00	0.89	1.13	0.94	0.08
E/e`	1.01	0.94	1.07	0.85	0.07
Global PACS (%)	1.13	0.71	1.79	0.62	0.07
E/A	1.11	0.68	1.84	0.67	0.06
Global PALS (%)	0.97	0.74	1.29	0.84	0.05
Beta-blocker	0.57	0.09	3.74	0.56	0.04
Digoxin	2.59	0.15	43.7	0.51	0.03
LA area (cm^2^)	0.99	0.92	1.07	0.83	0.03
Hypertension	1.09	0.37	3.28	0.87	0.02
Female sex	1.27	0.11	14.9	0.85	0.01
Other antiplatelet agent	0.93	0.22	3.97	0.92	0.01
Statin	0.95	0.31	2.93	0.93	0.01

Abbreviations as in Table 1 and Table 3

**Table 5:** Results of the multivariable regression model for prediction of secondary composite endpoint of death or hospitalization for ischemic stroke

	OR	−95% CI OR	95% CI OR	*p*-value
CHA_2_DS_2_-VASc score	1.7	1.2	2.5	0.006
ACE-I or ARB	0.2	0.05	0.8	0.02
BSA (m^2^)	0.02	0.0003	0.9	0.046

Abbreviations as in Table 1 and Table 3

## Discussion

The main finding of this study is that global PALS shows prognostic potential for LAAT identification in patients with HF, very low LVEF, and SR. PALS assessment could be a novel approach to non-invasive stratification of LAAT risk in this group of patients.

### Pathophysiological considerations

In healthy people, the lowest pressure in the LA is measured during the conduit phase [9], represented by PALS values. In the case of impaired LA wall extension, PALS is lower and LA pressure may be greater. This pressure is further increased as an effect of LV systolic dysfunction that results in impaired blood inflow into the LA [14]. The increase in LA volume promotes LA wall fibrosis and stiffness, further increasing both the pressure in the LA and the risk of LAAT formation [15]. Low PALS values reflects increased pulmonary capillary wedge pressure [16], and can predict reduced LA appendage emptying velocities [5].

### PALS values

LA function assessments could be performed using various methods; it is not clear whether TDI and speckle tracking technique results can be directly compared despite conveying analogous information [17]. Some authors use R-R gating instead of P-P gating, which moves the baseline to the nadir of contractile strain and increases PALS values when compared to P-P gating, as in our study (Fig. 1). As a result, reported absolute values of LA longitudinal strain could be as high as 20–30% in patients with mean LVEF < 35% [18]. In one study, in HF patients with LVEF < 35% and SR, global PALS values have been as high as 9.8% ± 4.2 and 16.9% ± 4.0% in patients with and without elevated pulmonary capillary wedge pressure, respectively [16]. The present findings in subjects with SR and LVEF < 25% were lower (average global PALS 9%), probably because of more severe depression in LV function and presumed higher LV filling pressures.

Our reliability and reproducibility analyses of TDI PALS showed very good intra-observer variability and moderately good inter-observer agreement. There is a significant learning curve associated with LV strain analysis by speckle tracking echocardiography [19]. Our analysis also suggests that although global PALS assessment by TDI is reproducible, an echocardiographist should have some experience in this method to produce more reliable measurements.

### LAAT prediction

LAAT is rarely present in patients with SR and normal LVEF [20, 21]. Nevertheless, in specific groups of patients, LAAT is more frequent. This includes patients with mitral stenosis, AF, atrial flutter, or LV systolic dysfunction [21–23]. A study in patients with AF showed that global PALS together with LA volume and increased E/e’ ratio was useful for predicting LAAT, while global PALS demonstrated the highest diagnostic accuracy [5]. The risk is greater in patients with LV systolic dysfunction regardless of heart rhythm [24–26]. Another study reported that LA deformation parameters measured by 2D speckle tracking predicted the presence of LAAT in ischemic stroke patients who were in SR [20]. Nevertheless, those patients had normal LVEF and only nine subjects with LAAT were identified. In the present study, we confirmed the prognostic value of PALS in predicting LAAT in patients with LV systolic dysfunction and SR. In our study, only two other echocardiographic variables (LAVI and global PACS) were significant predictors of LAAT in the univariate analysis; however, they convey less predictive information than does PALS and became obsolete in multivariate analysis once global PALS was included in the model.

In AF patients, a global PALS cut-off values less than 8.1% had good sensitivity and specificity to predict LAAT [5]. We found a similar value as an optimal cut-off point in patients with SR. This most likely suggests that global PALS of around 8% may be a strong independent predictor of LAAT, whether in SR or AF. In our study population, global PALS < 8% increased the risk of LAAT over 30-fold.

### Possible clinical implications

There is currently no recommendation for active screening for LAAT in the general population of HF patients. In our cohort of patients with very low LVEF (but without a diagnosis of AF) LAAT was present in 31.7%. This translates into a potentially large population of patients who may be candidates for antithrombotic treatment. Currently, the decision to initiate anticoagulation needs to be made after confirmation of LAAT using established imaging modalities. Nevertheless, PALS assessment may identify patients who might be candidates for such additional testing.

Routine use of anticoagulation in HF patients is currently not recommended [4, 27]; however, individualized decisions should be made, accounting for individual thromboembolic risks. The benefits of antithrombotic treatment in patients with severe LV systolic dysfunction and LAAT are rarely documented in clinical trials. Following current recommendations [4], we initiated anticoagulation if LAAT was confirmed using TEE. Therefore, we did not directly address the question of benefits of such therapy. The limited life expectancy of these severely ill patients with very low LVEF is another factor to be considered when translating LAAT presence into potential clinical implications.

Some clinical variables have been reported to have prognostic value for unfavorable clinical outcomes in HF patients in SR. These include hypertension, lower ejection fraction [1], prior stroke/TIA, and diabetes [3]. The assessment of LA reservoir strain provides additional risk stratification for stroke in AF patients [28], offering incremental value over the CHA_2_DS_2_-VASc score [29]. CHA_2_DSs_2_-VASc is a well-established risk score for thromboembolic complications and stroke in AF patients. Nevertheless, the usefulness of the CHA_2_DS_2_-VASc score in patients with SR is limited and its predictive accuracy for thromboembolic events or death appears only modest [30]. We found it to be the strongest predictor of our secondary composite endpoint of death or hospitalization for ischemic stroke. The positive impact of the use of ACE-I or ARB on clinical outcomes is not surprising given well-established benefits of these drugs in HF patients with reduced LVEF [27]. The borderline significance of BSA in our secondary endpoint analysis may reflect the so-called obesity paradox. It is well established that obesity is associated with lower mortality in HF patients, as is seen in other chronic illnesses [27]. In our study, neither PALS value nor LAAT had prognostic value in terms of the composite endpoint of all-cause mortality or hospitalization due to ischemic stroke. Nevertheless, LAAT is a potentially modifiable condition that is effectively treated with anticoagulants. In our study, the patients with LAAT had anticoagulation treatment initiated at the time of the diagnosis and stroke rates in this group could have been reduced, possibly explaining why we did not find a significant association between LAAT or PALS and our chosen clinical outcomes.

### Limitations

Women were underrepresented in our study. The patients were selected based on “real-life” clinical practice criteria – if they had no history of AF or AHRE nor other established indications for anticoagulation, they were considered eligible for the study. The study protocol did not require any intentional screening for AF and it was possible that some of the included patients experienced silent paroxysms of AF. Nevertheless, many of included patients had CIEDs and underwent regular cardiologic monitoring to allow detection of possible indications for anticoagulation. Furthermore, some of the included patients underwent Holter ECG monitoring during the index hospitalization as a part of routine clinical practice. If any of these modalities confirmed AF or AHRE, the patients were not included in the study. The actual ischemic stroke rates were low; however, this rate could be biased by the fact that patients in whom LAAT was identified were treated with anticoagulation agents. No data were collected regarding the adherence to the anticoagulant therapy nor information if the medications recommended at discharge were later changed or withdrawn. The lack of this data may affect the secondary endpoint interpretation. Further research is needed to determine whether the initiation of anticoagulation or additional screening supported by PALS measurements improves clinical outcomes in such patients.

## Conclusions

PALS has prognostic potential in LAAT identification in patients with HF, LVEF < 25%, and SR and without established indication for antithrombotic treatment. Global PALS values < 8% identify a group of patients with considerably higher risk of LAAT. LAAT was a relatively common finding in this group of HF patients.

## Data Availability

The datasets used and/or analysed during the current study are available from the corresponding author on reasonable request.

## Abbreviations

A2C: Apical two chamber
A4C: Apical four chamber
ACE-I: Angiotensin-converting-enzyme inhibitors
AF: Atrial fibrillation
AHRE: Atrial high-rate episodes
ARB: Angiotensin receptor blockers
AUC: Are under the ROC curve
BMI: Body mass index
BP: Blood pressure
BSA: Body surface area
CI: Confidence intervals
CIED: Cardiac implantable electronic devices
E/A: The ratio of early to late transmitral diastolic velocities
e’: Pulsed-wave TDI-derived mitral annular early diastolic velocity
ECG: Electrocardiography
HF: Heart failure
HR: Heart rate
ICC: Intraclass correlation coefficient
LA: Left atrium
LAA: Left atrium area
LAAT: Left atrial appendage thrombus
LAVI: Left atrial volume index
LV: Left ventricle
LVEF: Left ventricular ejection fraction
MRA: Mineralocorticoid receptor antagonist
NYHA: New York Heart Association
PACS: Peak atrial contraction strain
PALS: Peak atrial longitudinal strain
ROC: Receiver-operating characteristic
SEM: Standard error of measurement
SR: Sinus rhythm
TDI: Tissue Doppler imaging
TEE: Transesophageal echocardiography
TIA: Transient ischemic attack
TTE: Transthoracic echocardiography
